# A Quantum secure sharing protocol for Cloud data based on proxy re-encryption

**DOI:** 10.1038/s41598-020-65738-y

**Published:** 2020-06-03

**Authors:** Yan Chang, Shi-Bin Zhang, Li-Li Yan, Guo-gen Wan

**Affiliations:** School of Cybersecurity, Chengdu University of Information Technology, Chengdu, 610225 China

**Keywords:** Quantum information, Qubits

## Abstract

A quantum scheme for cloud data sharing based on proxy re-encryption is proposed. The user Alice stores the cipher-text of her data on cloud data center. When Alice wants to share her data with another user Bob, Alice is called the delegator and Bob is called the delegatee. The cloud service provider (called the proxy) can convert the delegator’s cipher-text into the delegatee’s cipher-text without decrypting the former, so that the delegatee can get the plain-text of Alice’s data with his private key. The proxy cannot obtain the plain-text of the user’s data stored on cloud data center. Delegator in the protocol should have the ability of producing Bell states, performing Bell basis and Z-basis measurements, and storing qubits. The quantum requirements for the delegatee are reduced. The delegatee needs to have the ability of reflecting and performing Z-basis measurement. One secret at a time (one-time one-pad) is theoretically implemented, especially when the same data is shared multiple times. The anti-selection plain-text attack security and the anti-selective cipher-text attack security are realized. Fine-granularity secret data sharing is achieved flexibly.

## Introduction

Proxy re-encryption is a kind of secret sharing method, but it is different from secret sharing in common meaning. In general, secret sharing^1^ refers to the split of secrets into several shares, and each share is managed by different participants. A single participant cannot recover secret information. Only a number of participants can work together to recover secret messages. Typical schemes are secret sharing schemes SSSs^2–4^ and multi-secret sharing schemes (MSSSs)^5–7^.

Proxy re-encryption is a new secret sharing method in cloud environment. The classical proxy re-encryption adds a proxy to the traditional public key encryption system. On the basis of the authorization of Alice (Alice give a conversion key to the proxy), the proxy can convert the cipher-text of Alice’s data into the cipher-text of Bob without decryption, and the proxy cannot obtain the plain-text of Alice’s data. This not only protects the key of Alice, but also ensures the security of Alice’s data. The concept of proxy re-encryption is proposed by Blaze, Bleumer and Strauss^8^ on Eurocrypt’98. In fact, proxy re-encryption does not need to re-encrypt, only the cipher-text is converted simply. Therefore, proxy re-encryptionis also called proxy conversion encryption. In 2005, on ACM CCS 2005, Ateniese, Fu, Green and Hohenberger gave the formal definition of their specification and proposed the first proxy re-encryption scheme^9^. This scheme is a two-way authorized proxy re-encryption scheme. That is, the proxy can transform not only the cipher-text of Alice’s data into the cipher-text of Bob, but also the cipher-text of Bob’s data into the cipher-text of Alice. Later, Ateniese, Fu, Green and Hohenberger proposed a one-way authorization proxy re-encryption scheme^10^. At the annual meeting of CCS 2007, Canetti and Hohenberger proposed a scheme of proxy re-encryption copywriting against selective cipher-text attack^11^. In 2008, Liber and Vergnaud proposed a one-way proxy re-encryption scheme against reproducing selected cipher-text attack^12^. In order to simplify the public key infrastructure in the proxy re-encryption scheme, Green and Ateniese proposed an identity-based proxy re-encryption scheme^13^ on the basis of the identity-based public key encryption scheme of Boenh and Franklin^14^. This scheme is proved to be safe under the random prophet model. Then Chu and Tzeng proposed a secure identity-based proxy re-encryption scheme without random prophet model^15^ based on identity-based public key encryption^16^. Weng, Deng and Chu put forward the concept of conditional proxy re-encryption^17^. In conditional proxy re-encryption scheme, only cipher-text that meets certain conditions can be re-encrypted by proxy. Subsequently, many conditional proxy re-encryption schemes^18–22^ and identity-based conditional proxy re-encryption schemes^23^ were proposed. In order to express the conditions and identities in conditional re-encryption more abundant, Liang, Cao, Lin and Shao proposed the concept of attribute-based proxy re-encryption^24^, and then many attribute-based conditional proxy re-encryption schemes^25–28^ were proposed.

With the rapid development of quantum technology, the schemes of quantum encryption^29–34^ and quantum secret sharing^35–41^ are emerging. However, there is no quantum proxy re-encryption protocol yet. In this paper, a proxy re-encryption protocol based on quantum carriers and quantum principle is proposed. Delegator in the protocol should have the ability of producing Bell states, performing Bell basis and Z basis measurements and storing qubits. While the delegatee is only need to have the ability of performing Z basis measurement and reflecting^33,34,38–40^, which reduces the quantum requirements for the delegatee, making it easier to implement. Proxy in the protocol can convert the cipher-text of the delegator (Alice) into the cipher-text of the delegatee (Bob) without decryption, and the proxy cannot obtain the corresponding plain-text information.

## The Protocol

### The goal of the protocol

**Figure 1:**
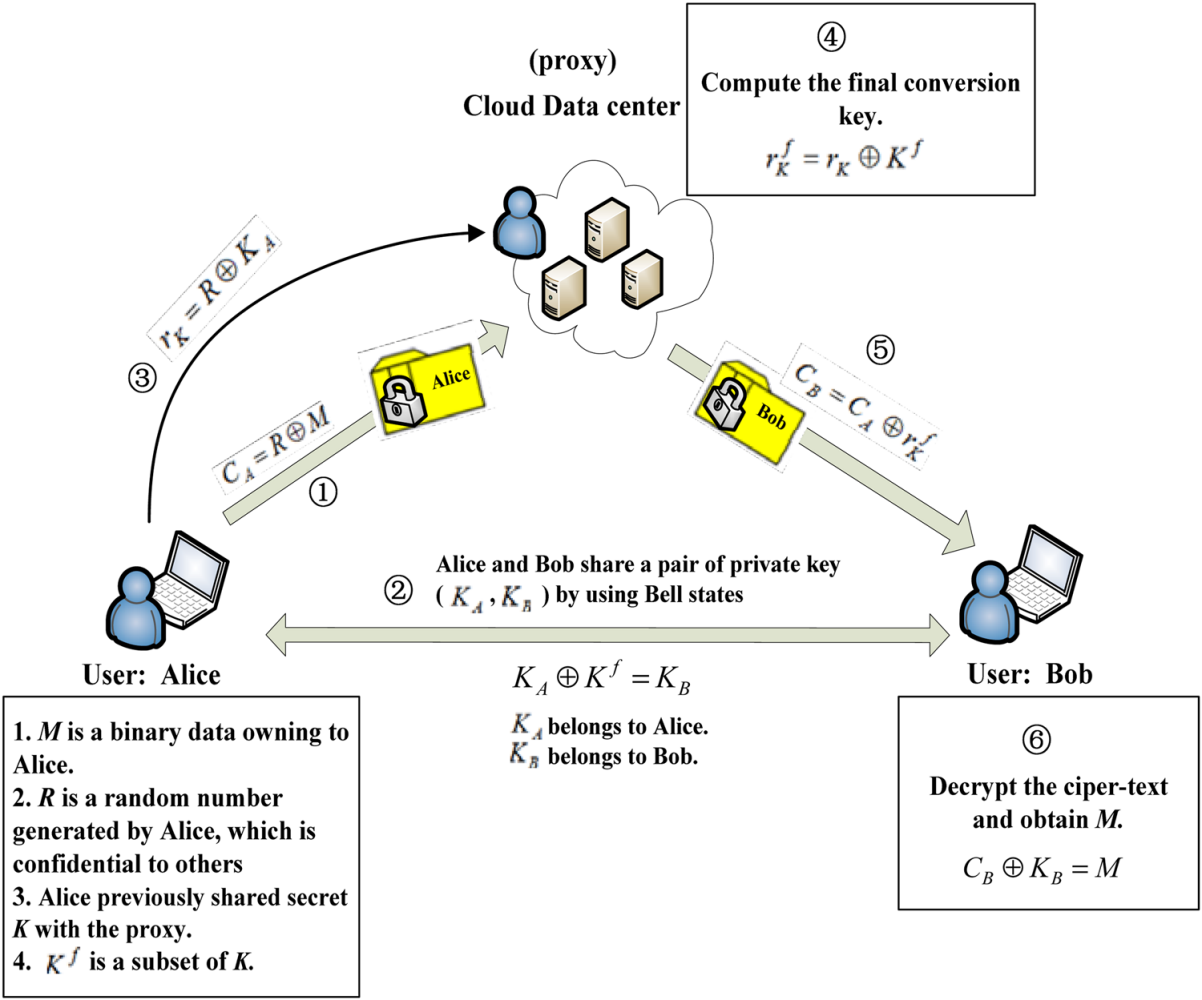
The structure of the protocol.

Alice and Bob are both users of a cloud data center. $M\in {\{0,1\}}^{n}$ is a binary data belonging to Alice. Alice stores the cipher-text of *M* on the cloud data center. The cloud service provider is called the proxy. The cipher-text of *M* is denoted as ${C}_{A}\in {\{0,1\}}^{n}$, where ${C}_{A}=M\oplus R$. $R\in {\{0,1\}}^{n}$ is a random number generated by Alice using quantum random number generator and is confidential to others. If Alice wants to share *M* with Bob, they can finish the task securely with the help of the proxy. The general process is as follows: Alice first sends a conversion key ${r}_{K}\in {\{0,1\}}^{n}$ to the proxy to let him generate the final conversion key ${r}_{K}^{f}\in {\{0,1\}}^{n}$. Then, the proxy uses ${r}_{K}^{f}$ to change the cipher-text *C*_*A*_ to Bob’s cipher-text ${C}_{B}\in {\{0,1\}}^{n}$. Bob decrypts *C*_*B*_ to get the plain-text *M* by using his private key ${K}_{B}\in {\{0,1\}}^{n}$. *K*_*B*_ can be obtained by executing the initial algorithm of the protocol, which will be described in the definition 3 of section 2.3. The proxy cannot know the plain-text *M*. The relation between *K*_*B*_ and other variables will be described in section 2.3. Figure 1 shows the whole structure of the protocol.

### Preliminaries

#### **Definition 1**:

Bell state is an important two-qubit state, which has four states:

1 $${\phi }^{\pm }=\frac{1}{\sqrt{2}}(|00\rangle \pm |11\rangle ),\,{\psi }^{\pm }=\frac{1}{\sqrt{2}}(|01\rangle \pm |10\rangle )$$

The Bell states ${\phi }^{\pm }=\frac{1}{\sqrt{2}}(|00\rangle \pm |11\rangle )$ have the property that upon measuring the first qubit, one obtains two possible results: 0 with probability 1/2, leaving the post-measurement state ${\phi }^{\pm }=|00\rangle$, and 1 with probability 1/2, leaving ${\phi }^{\pm }=|11\rangle$. As a result, a measurement of the second qubit always gives the same result as the measurement of the first qubit. That is, the measurement outcomes are correlated.

Similarly, the Bell states ${\psi }^{\pm }=\frac{1}{\sqrt{2}}(|01\rangle \pm |10\rangle )$ have the property that upon measuring the first qubit, one obtains two possible results: 0 with probability 1/2, leaving the post-measurement state ${\psi }^{\pm }=|01\rangle$, and 1 with probability 1/2, leaving ${\psi }^{\pm }=|10\rangle$. As a result, a measurement of the second qubit always gives the opposite result as the measurement of the first qubit. That is, the measurement outcomes are also correlated.

#### **Definition 2:**

Z-basis $\{|0\rangle ,|1\rangle \}$ measurement is the measurement of a qubit in the computational basis. This is a measurement on a single qubit with two outcomes defined by the two measurement operators ${M}_{0}=|0\rangle \langle 0|$, ${M}_{1}=|1\rangle \langle 1|$. The measurement operators satisfy the completeness. Suppose the state being measured is $|\psi \rangle =\alpha |0\rangle +\beta |1\rangle$. Then the probability of obtaining measurement outcome 0 is $p(0)={|\alpha |}^{2}$. Similarly, the probability of obtaining the measurement outcome 1 is $p(1)={|\beta |}^{2}$. The state after measurement in the two cases is therefore $|0\rangle$ or $|1\rangle$.

### Algorithm definition

When Alice’s data stored on cloud server is to be shared with Bob, Alice is the delegator, Bob is the delegatee, and the cloud server is the proxy. Alice previously shared $K\in {\{0,1\}}^{N}$ with the proxy by executing quantum key distribution protocol.

#### **Definition 3:**

The Initial Algorithm

Initial(*K*): On inputting the secret key $K\in {\{0,1\}}^{N}$, this algorithm works as below:

1. Alice prepares *N* Bell states according to *K*. The preparation rule is: ‘0’ to prepare state ${\phi }^{+}$ and ‘1’ to prepare state ${\psi }^{-}$.
2. Alice reserves one particle of each Bell state and sends the other particle to Bob.
3. Bob randomly performs Z-basis {|0〉, |1〉} measurement or reflecting on each particle he received. Bob saves the measurement results as ${K}_{B}\in {\{0,1\}}^{n}$, where ‘0’ denotes result |0〉, and ‘1’ denotes |1〉.
4. Alice performs joint Bell-basis measurements on reflected particles she received and the corresponding reserved particles. If each measurement result is consistent with the Bell state that originally prepared, or if the inconsistent ratio is below the predetermined threshold, the protocol will continue, otherwise the protocol will be terminated.
5. Alice records the positions as *Q* where she doesn’t receive particles, and measures the corresponding reserved particles with Z-basis. She saves the measurement results as ${K}_{A}\in {\{0,1\}}^{n}$, according to the rule: ‘0’ for state |0〉 and ‘1’ for state |1〉.

#### **Definition 4:**

Key Generation Algorithm

KeyGen(*K*_*A*_, *R*): On inputting Alice’s secret key *K*_*A*_ and a random number *R*, this algorithm outputs key ${r}_{K}\in {\{0,1\}}^{n}$, where ${r}_{K}=R\oplus {K}_{A}$.

#### **Definition 5:**

Re-Encryption Key Generation Algorithm

ReKeyGen(*r*_*K*_): On inputting the secret key *r*_*K*_, this algorithm works as below:

1. Alice computes ${r{\prime} }_{K}=Encryp{t}_{K}({r}_{K},Q)$ and sends ${r{\prime} }_{K}$ to the proxy (cloud server). Here $Encryp{t}_{K}()$ can be any symmetric encryption algorithm except for XOR.
2. The proxy decrypts ${r{\prime} }_{K}$ with *K* to obtain *r*_*K*_ and *Q*.
3. According to *Q*, the proxy extracts the corresponding bits in *K* to get ${K}^{f}\in {\{0,1\}}^{n}$.
4. The proxy computes ${r}_{K}^{f}={r}_{K}\oplus {K}^{f}=R\oplus {K}_{A}\oplus {K}^{f}=R\oplus {K}_{B}$, and obtains the final conversion key ${r}_{K}^{f}\in {\{0,1\}}^{n}$. Here, ${K}_{A}\oplus {K}^{f}={K}_{B}$ is obtained according to the property of Bell states.

#### **Definition 6:**

Encryption Algorithm

Encrypt(*R*, *M*): On inputting a random number *R* and plain-text *M*, this algorithm outputs the cipher-text ${C}_{A}\in {\{0,1\}}^{n}$, where ${C}_{A}=R\oplus M$.

#### **Definition 7:**

Re-Encryption Algorithm

ReEncrypt(${r}_{K}^{f}$, *C*_*A*_): On inputting the final conversion key ${r}_{K}^{f}$ and cipher-text *C*_*A*_, this algorithm outputs the re-encryption cipher-text ${C}_{B}\in {\{0,1\}}^{n}$, where ${C}_{B}={C}_{A}\oplus {r}_{K}^{f}$.

#### **Definition 8:**

Decryption Algorithm

Decrypt(*C*_*B*_, *K*_*B*_): On inputting Bob’s secret key *K*_*B*_ and cipher-text *C*_*B*_, this algorithm outputs the plain-text *M*.

**Figure 2:**
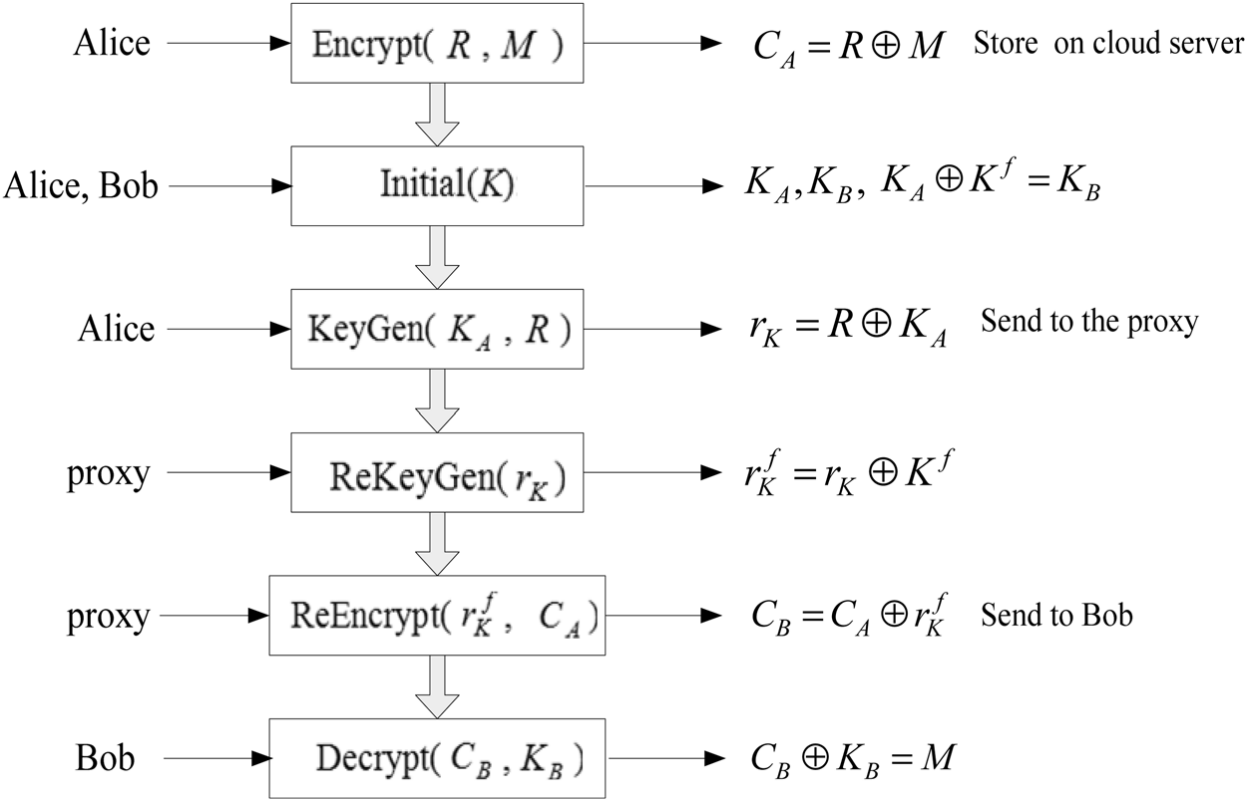
The process of algorithm execution.

Figure 2 shows the process of algorithm execution.

## The Security Proof of the Protocol

We conclude that the protocol satisfies the consistency according to the following derivation: Alice-> the proxy:

$$Encryp{t}_{K}({r}_{K},Q)$$

$${C}_{A}=R\oplus M$$

The proxy: $${r}_{K},Q=Decryp{t}_{K}(Encryp{t}_{K}({r}_{K},Q))$$

$${K}^{f}=Extract(K,Q)$$

$${r}_{K}^{f}={r}_{K}\oplus {K}^{f}$$

The proxy –> Bob: ${C}_{B}={C}_{A}\oplus {r}_{K}^{f}$

Bob: $${C}_{B}\oplus {K}_{B}={C}_{A}\oplus {r}_{K}^{f}\oplus {K}_{B}=R\oplus M\oplus {r}_{K}^{f}\oplus {K}_{B}=R\oplus M\oplus R\oplus {K}_{B}\oplus {K}_{B}=M$$

Generally speaking, in order to prove the security of a classical cryptography scheme, the security objectives are first determined. Then an attack model is constructed according to the ability of the attacker. Finally, the method specification for breaking the scheme is proposed to solve a difficult mathematical problem or difficult assumption.

In our scheme, Alice’s data is encrypted with a random number *R* and stored on cloud server. Any user that Alice is willing to share data with can realize the sharing by executing the protocol. The principles of quantum non-cloning, uncertainty and entanglement ensure that the re-encryption cipher-text of the same shared data in each sharing process is different, which means that one secret at a time (one-time one-pad) is realized. Therefore, it can be proved that the protocol can resist anti-selective plain-text attack and anti-selective cipher-text attack without using the classical reduction method.

The principles of quantum non-cloning, uncertainty and entanglement work on the premise that the protocol has the ability to discover or prevent attackers from falsifying quantum carriers. Section 4.1 and 4.2 prove that if the eavesdropper intends to falsifying quantum carriers (replacing or destructing the Bell states), his behavior will be found with very high probability (almost 99.9%).

Furthermore, in the protocol, the proxy only knows *K*^*f*^ (the entanglement relationship between *K*_*A*_ and *K*_*B*_), he cannot know the data stored on cloud server. Bob only know *Q* and *K*_*B*_, he does not know the relationship between *K*_*A*_ and *K*_*B*_, therefore he cannot know the data stored on cloud server without the re-encryption of cipher-text by the proxy.

## Security Analysis

### Intercept-resend attack

The external attacker Eve may intercept the particles that Alice sent to Bob, and measure them with Z-basis, then prepare some particles with the same state and send them back to Bob. Suppose that each particle reserved by Alice is expressed as particle 1, each particle sent to Bob is represented as particle 2, and each particle re-prepared by Eve is represented as particle *e*. Then, after Eve intercepting and measuring particles 2 with Z-basis, the state of particle 1 collapses to ${\rho }_{1}=\frac{1}{2}|0\rangle \langle 0|+\frac{1}{2}|1\rangle \langle 1|$. The state of the particle reflected by Bob is ${\rho }_{e}=\frac{1}{2}|0\rangle \langle 0|+\frac{1}{2}|1\rangle \langle 1|$. The combined state of particle 1 and particle *e* is:

2 $${\rho }_{1e}=\frac{1}{4}|00\rangle \langle 00|+\frac{1}{4}|11\rangle \langle 11|+\frac{1}{4}|01\rangle \langle 01|+\frac{1}{4}|10\rangle \langle 10|$$

If the initial combined state of particle 1 and 2 is ${\psi }^{-}$, after eavesdropping detection, the joint Bell-basis measurement result on particle 1 and *e* is as follows:

3 $${\rho {\prime} }_{1e}=\frac{1}{2}|{\psi }^{+}\rangle \langle {\psi }^{+}|+\frac{1}{2}|{\psi }^{-}\rangle \langle {\psi }^{-}|$$

If the initial combined state of the particles 1 and 2 is ${\phi }^{+}$, after eavesdropping detection, the joint Bell-basis measurement result on particle 1 and *e* is as follows:

4 $${\rho {\prime} }_{1e}=\frac{1}{2}|{\phi }^{+}\rangle \langle {\phi }^{+}|+\frac{1}{2}|{\phi }^{-}\rangle \langle {\phi }^{-}|$$

Therefore, Alice can discover Eve’s eavesdropping on each qubit with probability 1/2, and the total probability that Alice can detect Eve’s eavesdropping is 1 − (1/2)^*n*^. When *n* = 5, the probability reaches 97%. The protocol will be terminated, and the eavesdropper will not obtain any data that Alice stored on the cloud server.

### Source untrusted attack

The reflected particles are used for eavesdropping detection, not only detecting the intercept-resend attack, but also detecting the source untrusted attack^42–44^. Usually, in source untrusted attack, the eavesdroppers with super ability will control or provide devices used to prepare Bell states. Although Alice thinks a real Bell state is prepared, what she actually gets may be a different state because the preparing device is controlled or provided by Eve^42–44^. That is, the source is untrusted.

To steal secret message, Eve may control the device to prepare some non-entangled mixed states of |00〉, |01〉, |10〉, |11〉 or entangled states with higher dimensional such as GHZ states.

1. Eve prepares state ${\rho }_{12}=\frac{1}{2}|00\rangle \langle 00|+\frac{1}{2}|11\rangle \langle 11|$ instead of ${\phi }^{+}$ and prepares state ${\rho }_{12}=\frac{1}{2}|01\rangle \langle 01|+\frac{1}{2}|10\rangle \langle 10|$ instead of ${\psi }^{-}$. By doing so, Eve will know *K*_*A*_ and *K*_*B*_ before eavesdropping detection. However, during the eavesdropping detection, Alice performs the joint Bell-basis measurement on particles 1 and 2, and the following results will be obtained respectively:
  5 $${\rho {\prime} }_{12}=\frac{1}{2}|{\phi }^{+}\rangle \langle {\phi }^{+}|+\frac{1}{2}|{\phi }^{-}\rangle \langle {\phi }^{-}|\,{\rm{or}}\,{\rho {\prime} }_{12}=\frac{1}{2}|{\psi }^{+}\rangle \langle {\psi }^{+}|+\frac{1}{2}|{\psi }^{-}\rangle \langle {\psi }^{-}|$$
  Obviously, Alice will discover Eve’s eavesdropping on each qubit with probability 1/2, and the total probability that Alice finds Eve’s eavesdropping is $1-{(1/2)}^{n}$. Thus, the protocol will be terminated, and the eavesdropper will not obtain any data that Alice stored on the cloud server.
2. Eve prepares entangled state *G*_0_, *G*_1_ or ${\rho }_{123}=\frac{1}{2}|{G}_{0}\rangle \langle {G}_{0}|+\frac{1}{2}|{G}_{1}\rangle \langle {G}_{1}|$ instead of ${\phi }^{+}$, and prepares entangled state *G*_2_, *G*_3_ or ${\rho }_{123}=\frac{1}{2}|{G}_{2}\rangle \langle {G}_{2}|+\frac{1}{2}|{G}_{3}\rangle \langle {G}_{3}|$ instead of ${\psi }^{-}$. Here,

6 $$\begin{array}{c}{G}_{0}=\frac{1}{\sqrt{2}}{(|000\rangle +|111\rangle )}_{123},\,{G}_{1}=\frac{1}{\sqrt{2}}{(|001\rangle +|110\rangle )}_{123}\\ {G}_{2}=\frac{1}{\sqrt{2}}{(|010\rangle +|101\rangle )}_{123},\,{G}_{3}=\frac{1}{\sqrt{2}}{(|100\rangle +|011\rangle )}_{123}\end{array}$$

Eve sends particle 1 and 2 to Alice, and keeps particle 3 herself. When Bob measures the received particle 2 with Z-basis, the state of particle 1 and 3 collapse. Since Eve does not know on which positions Bob will measure and which positions to reflect, Eve will not measures those particles 3 on the un-reflected positions with Z-basis until she determines which positions are reflected. Although, by doing so, she will obtain *K*_*A*_ and *K*_*B*_, but before that, to detect eavesdropping Alice performs joint Bell-basis measurement on particle 1 and 2. If Eve prepares entangled state *G*_0_, *G*_1_ or ${\rho }_{123}=\frac{1}{2}|{G}_{0}\rangle \langle {G}_{0}|+\frac{1}{2}|{G}_{1}\rangle \langle {G}_{1}|$ instead of ${\phi }^{+}$, the measurement result is:

7 $${\rho {\prime} }_{12}=\frac{1}{2}|{\phi }^{+}\rangle \langle {\phi }^{+}|+\frac{1}{2}|{\phi }^{-}\rangle \langle {\phi }^{-}|$$

If Eve prepares entangled state *G*_2_, *G*_3_ or ${\rho }_{123}=\frac{1}{2}|{G}_{2}\rangle \langle {G}_{2}|+\frac{1}{2}|{G}_{3}\rangle \langle {G}_{3}|$ instead of ${\psi }^{-}$, the measurement result is:

8 $${{\rho }_{12}}^{{\prime} }=\frac{1}{2}|{\psi }^{+}\rangle \langle {\psi }^{+}|+\frac{1}{2}|{\psi }^{-}\rangle \langle {\psi }^{-}|$$

Obviously, before Eve knows *K*_*A*_ and *K*_*B*_, Alice will discover the eavesdropping behavior of Eve with probability $1-{(1/2)}^{n}$. Thus, the protocol will be terminated, and the eavesdropper will not obtain any data that Alice stored on the cloud server.

### Proxy attack

In this protocol, an honest proxy knows only the correlation between *K*_*A*_ and *K*_*B*_, but does not know exactly what *K*_*A*_ and *K*_*B*_ are. Therefore, the honest proxy cannot know *M* through ${C}_{B}=M\oplus {K}_{B}$. In addition, only the delegator knows the random number *R* which encrypted shared data *M*, so the proxy cannot know *M* through ${C}_{A}=R\oplus M$.

If the proxy is dishonest, assuming that he is the eavesdropper discussed in 4.1 and 4.2, besides having the power of eavesdroppers, he knows *K*. When the proxy performs intercept-resend attacks, having *K* will not help him with the success of his attack. Therefore, when Alice detects eavesdroppers, the attack will be found by Alice with probability $1-{(1/2)}^{n}$. And the proxy cannot know the shared data that Alice stored on the cloud server.

For an honest proxy, although he has the conversion key *r*_*k*_ and the final conversion key ${r}_{K}^{f}$, he cannot obtain the plain-text of shared data stored on the cloud server. For a dishonest proxy, his bad behavior will be detected with probability closing to 100%. Therefore, neither honest proxy nor dishonest proxy have access to the plain-text of shared data stored on the cloud server.

## The Comparisons with Previous Works

Compared with the previous classical proxy re-encryption protocols proposed in refs. ^8–12^, our protocol theoretically implements one secret at a time (one-time one-pad), especially when the same data is shared multiple times. In each data sharing process, *K*_*A*_, *K*_*B*_ and ${r}_{K}^{f}$ are random numbers with entanglement correlation, which is ensured by the principles of quantum non-cloning, uncertainty and entanglement. The second layer cipher-text (cipher-text of the delegatee) will not reappear. Therefore, the protocol realizes the anti-selection plain-text attack security and the anti-selective cipher-text attack security without basing on the difficult mathematical problem or difficulty assumption.

Compared with the protocols proposed in refs. ^8–16^, our protocol can flexibly achieve fine-granularity secret data sharing. Alice can control the sharing granularity to Bob by adjusting ${r}_{K}$ and the starting location of shared data. However, the protocol cannot resist the conspiracy attack of the proxy and Bob.

Our protocol requires Alice have the ability of producing Bell states, performing Bell basis and Z basis measurements and storing qubits. The quantum ability of Bob is low; he is only need to have the ability of performing Z basis measurement and reflecting. Compared with QSS protocols proposed in refs. ^37,39–41,45^, our protocol reduces the difficulty of implementation. In refs. ^39–41^, multi-particle entanglement states need to be prepared, which is more difficult than preparing Bell states. In ref. ^45^, although both classical and quantum secret sharing are designed, however the quantum Fourier transform and d-level quantum system are needed, which are more complex and difficult to implement than our protocol.

## Discussion

Smooth entropy and mutual information are usually used to analyse the security of quantum key distribution, i.e. secret key agreement by communication over a quantum channel^50–52^. In this section, we analyze the post-processing of the protocol from the perspective of mutual information.

In order to make the key shared by Alice and Bob logically consistent, and to reduce the amount of information Eve knows, the protocol has to carry out error reconciliation and privacy amplification. Eve may intercept the particle 2 sent by Alice to Bob, then measures it with the Z-basis and sends it back to Bob. Normally, Bob randomly chooses to reflect the particle or measure the particle with Z-basis. The results of the Z-basis measurement are taken as *K*_*B*_. Eve’s attack will not result in a bit error because the measurement basis is the same with Bob’s.

Let the bit error rate be *λ* for the environmental factors^46,47^ other than Eve’s above attack. In order to correct errors, at least the extra information of ${H}_{2}(\lambda )$ needs to be transmitted for each bit. After privacy amplification, the security key rate is:

9 $$r\le I(B:A)-I(B:E)=1-{H}_{2}(\lambda )-I(B:E)$$

Because the eavesdropping detection of the protocol is to detect whether the two parties share the entangled state ${\phi }^{+}$ or ${\psi }^{-}$, once the shared entangled state is confirmed by the eavesdropping detection, Eve cannot obtain the information of *K*_*B*_ according to the monogamy of nonlocal correlations (entanglement). Therefore, in our protocol, $I(B:E)=0$_._

10 $$r\le 1-{H}_{2}(\lambda )$$

Assuming that the length of the secret data *M* is *n*, the length of ${r}_{K}^{f}$, *K*_*A*_ and *K*_*B*_ must be *n* bits in order to ensure that the secret data can be successfully shared. Therefore, the length of ${r}_{K}^{f}$, *K*_*A*_ and *K*_*B*_ before error reconciliation and privacy amplification which is denoted as *m* must satisfy the following inequality

11 $$m\ge \frac{n}{1-{H}_{2}(\lambda )}$$

The number of Bell states prepared in the initial algorithm should satisfy:

12 $$N=2m\ge \frac{2n}{1-{H}_{2}(\lambda )}$$

## Conclusion

The proposed quantum cryptography^48,49^ protocol realizes secure data sharing on cloud server based on proxy conversion encryption. In the protocol, the intercept-resend attack, the source untrusted attack, and the proxy attack are analyzed. Delegator in the protocol should have the ability of producing Bell states, performing Bell basis and Z basis measurements and storing qubits. While the quantum requirements for the delegatee are reduced. The delegatee is only need to have the ability to reflect and performing Z-basis measurement, which satisfies the semi-quantum condition. In data loss scenario, to ensure the normal keys sharing, after Alice sends particles to Bob, Bob should publish which particles are lost, and Alice discards the corresponding particles. Alice and the proxy should discard the corresponding bits of *K* before extracting *K*^*f*^.
